# Ultrasound-Assisted Solid-Phase Affibody Synthesis Using Z_EGFR:1907_ as an Example—Superior to the Conventional Protocol?

**DOI:** 10.3390/ph17101280

**Published:** 2024-09-27

**Authors:** Marie Prochiner, Benedikt Judmann, Alina Ruder, Björn Wängler, Ralf Schirrmacher, Carmen Wängler

**Affiliations:** 1 Biomedical Chemistry, Clinic of Radiology and Nuclear Medicine, Medical Faculty Mannheim, Heidelberg University, Theodor-Kutzer-Ufer 1-3, 68167 Mannheim, Germany; marie.prochiner@stud.uni-heidelberg.de (M.P.); benedikt.judmann@medma.uni-heidelberg.de (B.J.); ruderal102168@th-nuernberg.de (A.R.); 2 Research Campus M²OLIE, Medical Faculty Mannheim, Heidelberg University, Theodor-Kutzer-Ufer 1-3, 68167 Mannheim, Germany; bjoern.waengler@medma.uni-heidelberg.de; 3 Molecular Imaging and Radiochemistry, Clinic of Radiology and Nuclear Medicine, Medical Faculty Mannheim, Heidelberg University, Theodor-Kutzer-Ufer 1-3, 68167 Mannheim, Germany; 4 Division of Oncological Imaging, Department of Oncology, University of Alberta, Edmonton, AB T6G 1Z2, Canada; schirrma@ualberta.ca

**Keywords:** affibody, Z_EGFR:1907_, solid-phase peptide synthesis, ultrasonication, mechanical shaking

## Abstract

Background: Affibody molecules represent a class of highly specific binders of particular interest for the development of highly affine target-specific radiopharmaceuticals. Their chemical synthesis is, however, intricate due to their considerable length of 58 amino acids; thus, approaches to optimize their preparation are constantly being sought. Methods: As ultrasound assistance has recently been shown to increase the efficiency of amino acid conjugation during solid-phase peptide synthesis (SPPS), the influence of ultrasonication on the outcome of the SPPS-based preparation of the EGFR-specific affibody Z_EGFR:1907_ was compared to a common protocol relying on mechanical shaking. Results: After the identification of a suitable solid support for the study, the execution of the systematic comparison of both approaches showed that conventional and ultrasound-assisted syntheses yielded equivalent results with analogous composition of the raw products. Further, both approaches produced the affibody in good isolated yields of >20% when applying the same optimal reagent excesses and coupling times for the conjugation of each amino acid. This indicates that, under optimal reaction conditions, the choice of solid support used has a much stronger influence on the outcome of the preparation of Z_EGFR:1907_ than the application of ultrasound, which did not further improve the synthesis results. Conclusions: Therefore, for the chemical synthesis of affibodies, great attention should be paid to the choice of a suitable solid support, enabling this highly interesting class of biomolecules to be obtained in good yields and to bring them more into the focus of radiopharmaceutical research.

## 1. Introduction

The target-specific imaging of malignant lesions by combined Positron Emission Tomography/Computed Tomography (PET/CT) using positron-emitter-labeled substances has been standard clinical practice for many years [1,2]. In particular, peptides and antibodies are used as target-specific carriers for radioisotopes as they can achieve high target affinity and specificity, enabling high tumor-imaging sensitivity and specificity. Peptides, in particular, have become a valuable basis for the development of target-specific radioligands as, unlike high-affinity antibodies in the IgG format, they exhibit rapid pharmacokinetics with fast tumor uptake and non-target tissue clearance as well as efficient tissue penetration. However, there is still a lack of availability of highly specific and affine peptide scaffolds for every target structure, which can serve as a suitable basis for the development of corresponding radiopharmaceuticals.

One example in this context is the Epidermal Growth Factor Receptor (EGFR, also denoted as HER1), which is highly expressed in numerous tumor entities and, therefore, represents an important target structure for specifically accumulating radioligands [3,4,5]. However, despite several promising attempts, no peptide has yet been identified as a suitable basis for the development of EGFR-specific radiotracers [6,7,8,9]. Cetuximab, an antibody in the IgG format, is known to be a highly specific and affine-targeting vector for the EGFR, but, due to its format, it exhibits the disadvantages mentioned above.

**Figure 1:**
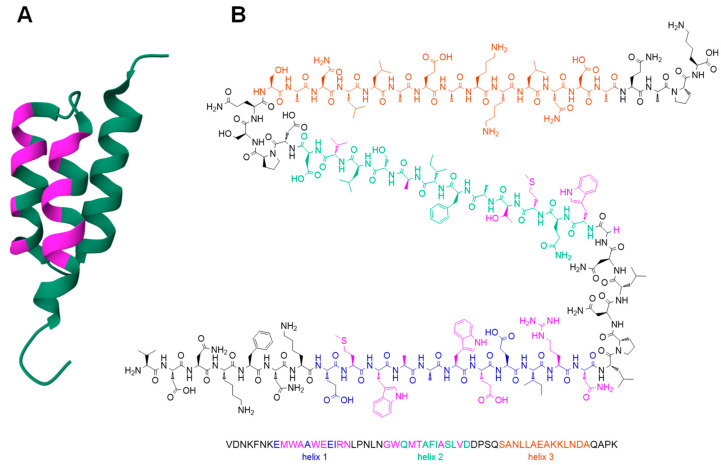
Depiction of the general tertiary structure of affibody molecules using the example of an HER2-specific affibody (Z_HER2_, structure and image generated in RCSB Protein Data Bank, PDB-ID 2KZJ) with the 13 variable, solvent-exposed residues depicted in pink (**A**). Structure of the affibody Z_EGFR:1907_ with the amino acids forming the helices 1, 2, and 3, colored in blue, green, and orange, respectively. The variable, solvent-exposed amino acids responsible for EGFR-specific binding are highlighted in pink (**B**).

An alternative to full-length antibodies addressing the EGFR could be affibodies directed against this receptor type, as they exhibit more favorable pharmacokinetics than full-length antibodies. Affibodies are composed of 58 amino acids, are regarded as small proteins that spontaneously and stably arrange in a tri-helical structure, and are derived from staphylococcal protein A. By varying the 13 solvent-exposed residues (Figure 1A), highly specific binders against various target structures can be obtained by means of affinity maturation [10]. Such affibody structure optimizations, amongst others, have been carried out for the EGFR and high-affinity binders for this receptor type have been generated using this approach [11].

One of the most promising of these is the affibody Z_EGFR:1907_ (Figure 1B), which exhibits a high affinity to the EGFR (K_d_ of 5.4 nM [12]) and has already been used in ^18^F- and ^64^Cu-labeled form for the imaging of EGFR-expressing human tumor cells in xenografted mice [13,14].

However, the use of affibodies has significant limitations compared to short peptides, which are conventionally used as basis for radiopharmaceuticals [15]. Their synthesis by conventional chemical methods is complex due to the many necessary amino acid conjugation steps, all of which have a high probability of yielding the desired product, but also a low likelihood of producing side reactions. This can make it difficult to impossible to isolate the target molecule from the complex product mixture, as defects accumulate over the course of the synthesis. The formation of these defects can be due to various causes such as the incomplete removal of the protecting group, incomplete reaction with the following building block, racemization, and aspartimide formation [16]. The incomplete conjugation of the synthesis building blocks results in truncated products that are, however, equally capable of further reaction as the intended products. The aim is, therefore, to achieve the highest possible reaction efficiency for each individual coupling reaction step.

In order to maximize reaction efficiencies during SPPS, mechanical shaking is often employed to avoid the depletion of the reagents on the functional group immobilized on the solid phase. Although this reduction of concentration gradients also occurs through diffusion, this effect is only capable of reducing the arising gradients to a limited extent, as the time required to achieve complete homogenization increases with the square of the distance and is, therefore, limited to short distances. Further, mixing can not only be achieved by mechanical shaking, but also by stirring and gas bubbling [17,18]. Heating [19] or ultrasonication [20] can also be used to accelerate the equalization of concentration differences.

An alternative to the conventional linear synthesis strategy of peptides via SPPS is the convergent synthesis of several peptide fragments, which can be ligated to the final intended product by either standard acid–amide coupling (but requiring the synthesis of fully protected peptide fragments to avoid side reactions during conjugation and long reaction times to enable the complete reaction of such bulky peptide fragments) or native chemical ligation (NCL) techniques [21]. The latter rely on the use of thiol-containing building blocks for the particular ligation reactions and either result in the formation of cysteines in the peptide sequence (which are not present in natural affibody molecules and, thus, the biological testing of the molecules is necessary to ensure unaffected target affinity and specificity) [22] or necessitate subsequent desulphurization steps, which often require some optimization [23].

The alternative to the chemical synthesis of affibodies is their recombinant production; however, this does not allow the regiospecific derivatization of the proteins with artificial scaffolds such as chelating agents used for radiolabeling with radiometal nuclides. However, this site-specific modifiability is usually a prerequisite for obtaining radiolabeled biomolecules with high target affinity.

Strategies for the optimization of the chemical synthesis of affibodies are, therefore, constantly being sought. For example, attempts have been made to improve the individual conjugation steps of affibody SPPS using microwave irradiation or heating, but both approaches resulted in decreased product purities compared to conventional, non-microwave-assisted syntheses at ambient temperature [24]. This can most likely be attributed to the fact that heating can immensely improve the synthesis results during SPPS, especially when reduced coupling efficiencies are a result of sequence aggregation that can be overcome by heating, but can, on the contrary, also promote racemization and aspartimide formation [19].

In recent years, another peptide preparation procedure for improving SPPS yields and efficiency has moved into the focus of research, namely, ultrasound-assisted synthesis. The ultrasonication of the reaction mixtures was demonstrated to positively influence amino acid building block coupling efficiencies under SPPS conditions for peptides of up to 44 amino acids in length [6,20,25] and, thus, could be a very promising approach—especially with regard to the long amino acid sequence of affibodies—to significantly improve the Fmoc-strategy-based SPPS of this class of bioactive compounds.

The aim of this work was, therefore, to investigate, using the example of the above-mentioned affibody Z_EGFR:1907_, whether this approach is actually suitable in achieving superior synthesis results compared to the conventional procedure of applying mechanical shaking during the individual SPPS conjugation steps.

## 2. Results

Before the systematic evaluation and comparison of the ultrasound-assisted against the conventional mechanical-shaking-based SPPS of Z_EGFR:1907_ was conducted, preceding tests were carried out to determine a suitable solid support for the synthesis of 58-amino-acid-comprising affibodies. For this purpose, two conventional low-loading resins—a Sieber TG resin with a loading of 0.21 mmol/g and a Rink resin with a loading of 0.36 mmol/g—were used initially, as they are often used in the synthesis of complex peptide sequences. The affibody was assembled on these solid supports by successive conjugation of the respective *N*_α_-Fmoc- and side-chain-protected amino acid building blocks that had been activated using HBTU (2-(1*H*-benzotriazol-1-yl)-1,1,3,3-tetramethyluronium hexafluorophosphate) and DIPEA (*N*,*N*-diisopropylethylamine) as the base. The product was finally cleaved from the resin and simultaneously side-chain-deprotected under acidic conditions. These investigations were initially carried out using different excesses of reagents and testing different reaction times, but the results—presumably due to these non-optimal reaction conditions—varied considerably. Therefore, for the systematic investigations carried out later, we decided to apply optimal reaction conditions with sufficiently long reaction times and high excesses (vide infra). The results obtained showed very little variance and high reproducibility. Using the mentioned resins for the affibody synthesis under common SPPS conditions, marked differences were found between the products obtained from both resins when analyzing the raw product mixtures after peptide cleavage and deprotection at different stages of the syntheses by analytical HPLC (high-performance liquid chromatography) combined with MALDI mass spectrometry (MALDI: matrix-assisted laser desorption/ionization). When applying the Rink acid resin, the intended product could not be detected, but the longest fragment that could be produced using this solid support was a 45-amino-acid-comprising sequence. Thus, the Rink resin seemed to be of limited utility for the synthesis of EGFR-specific affibodies. In contrast, product formation was observed using the Sieber TG resin. Here, considerable differences in product mixture composition were found when comparing the conventional and ultrasound-assisted synthesis techniques. While the product was detectable in the case of the application of the conventional synthesis protocol (even if it only made up a very small proportion of the raw product mixture), the purity of the crude product was even lower when ultrasonication was used to increase the conjugation efficiency (Figure S1). As the intended product could barely be analyzed or even isolated due to the large number of by-products formed, further attempts using these two solid supports were omitted as none of them was found to be suitable for the SPPS of complex affibody molecules.

As a consequence, we switched the solid support to a NovaSyn TGR R resin, as this material was described by the manufacturer to exhibit improved swelling properties compared to a conventional NovaSyn TGR resin and was specifically designed for the synthesis of extra-long peptide sequences comprising more than 50 amino acid residues. Thus, the NovaSyn TGR R resin was used to conduct the comparative systematic study on the influence of ultrasound assistance on affibody product formation during SPPS.

In order to be able to investigate the influence of ultrasonication alone on the synthesis results of the Z_EGFR:1907_ affibody, all other reaction parameters such as excesses of amino acids (4 eq. of *N*_α_-Fmoc- and side-chain-protected amino acid), coupling agent (3.9 eq. of HBTU) and base (4 eq. of DIPEA) used for activation were kept identical for both approaches. Also, the building block coupling times were kept constant with 30 min for amino acids 1–20, 45 min for amino acids 21–40, and 60 min for amino acids 41–58. These conditions translated into an overall total time of about 70 hours per affibody synthesis as the sum of time required for the individual steps of the preparation: resin swelling, amino acid building block activation and coupling, Fmoc cleavage reactions after each conjugation step, washing steps, and final acidic cleavage from the solid support. Three replicates were made per condition (conventional and ultrasound-assisted) and after each coupling of 10 amino acids, a small portion of each batch was cleaved from the solid support and analyzed by HPLC and mass spectrometry to determine sequence or purity differences between both approaches at different stages of the synthesis.

**Figure 2:**
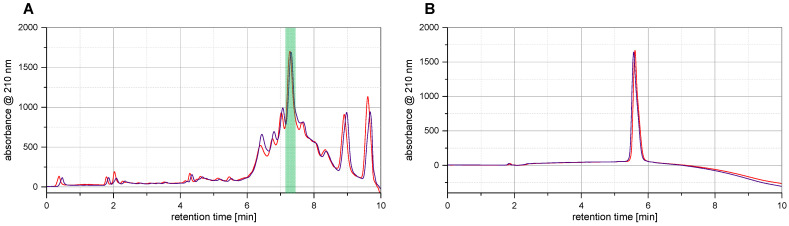
(**A**) Comparison of the HPLC analyses of the crude product mixtures after cleavage of the complete affibody sequence **6** under both conditions applied (violet: crude product obtained by ultrasound-assisted coupling steps, red: crude product obtained by conventional coupling based on mechanical shaking) using semi-preparative HPLC and a gradient of 10–60% MeCN over 8 minutes. The intended product **6** is highlighted in green in the chromatograms. (**B**) HPLC chromatograms of purified **6** (violet: **6** obtained by the ultrasound-assisted coupling protocol, red: **6** obtained by the conventional coupling protocol) using semi-preparative HPLC and a gradient of 0–100% MeCN over 8 minutes. UV signals were normalized for better comparability of the chromatograms.

Under the mentioned conditions, we found the NovaSyn TGR R resin to be, indeed, much better suited for the synthesis of long-peptide sequences compared to the resins tested before, as significantly more of the desired product, Z_EGFR:1907_ (**6**), was detectable in the crude with lower quantities of by-products formed. As a result, the intended affibody molecule became the predominant product in the raw mixture with a proportion of about 25% after cleavage and deprotection, with fewer by-products compared to previous methods where the product accounted for <7% of the crude product (Figure 2).

**Figure 3:**
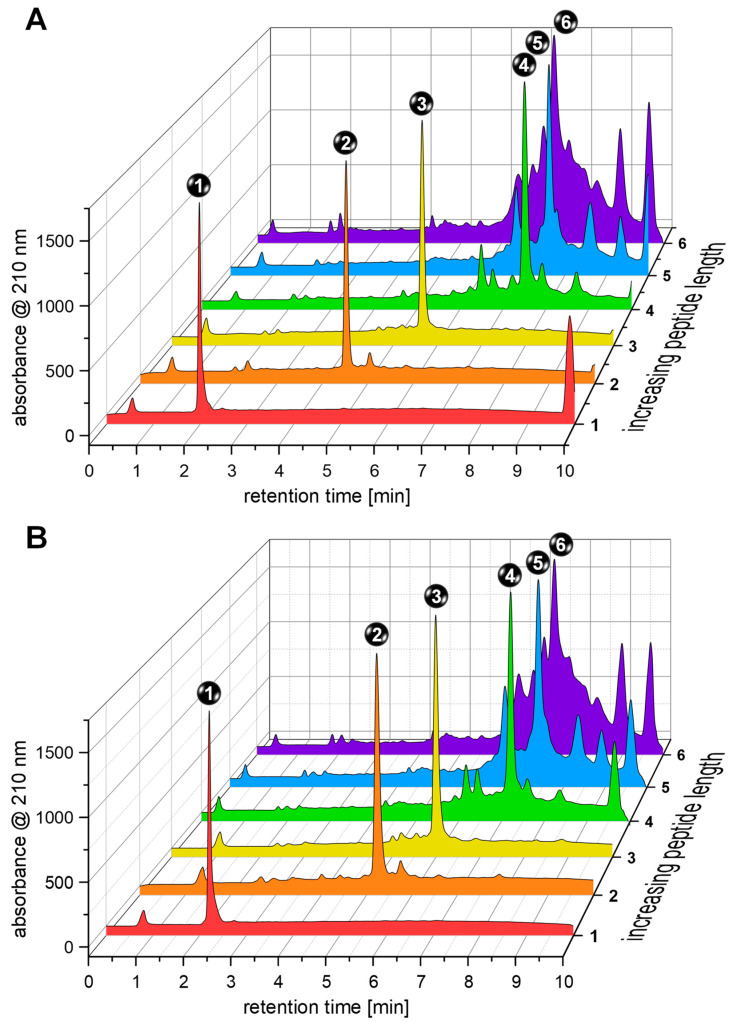
Direct comparison of the HPLC analyses conducted on the peptide fragments **1**–**5** (**1**: amino acids 49–58, **2**: amino acids 39–58, **3**: amino acids 29–58, **4**: amino acids 19–58, and **5**: amino acids 9–58) and the full-length affibody sequence **6** (amino acids 1–58) obtained by applying (**A**) conventional mechanical-shaking-based and (**B**) ultrasound-assisted synthesis conditions. The UV signals were normalized for better comparability of the chromatograms.

Both approaches—conventional mechanical-shaking-based and ultrasound-assisted syntheses—gave highly comparable results regardless of the stage of the synthesis and resulted in the formation of the intended product as the main component of the respective mixture (Figure 3 and Table 1). It can be observed that the purity of the target compound was high up to and including the 30th coupling step, decreased considerably up to the coupling of the 50th amino acid, and further declined beyond the 50th coupling step (Figure 3) regardless of the applied conditions (conventional or ultrasound-assisted).

Over the course of the synthesis, the proportion of the respective intended product (fragment or full-length affibody) in the crude mixtures decreased with the number of coupled amino acid building blocks from 91.4 ± 2.2% to 24.8 ± 2.4% for the conventional synthesis and from 94.7 ± 3.0 to 25.3 ± 3.2% for the ultrasound-assisted synthesis protocol, thus not demonstrating significant differences between the two approaches. Considering the isolated product yields of **6**, these were also well comparable with 24.4 ± 0.6% for the conventional and 20.5 ± 6.1% for the ultrasound-assisted syntheses (Table 1).

It should be mentioned at this point that isolated product yields of more than 20% are very satisfactory for peptides of this size and complexity and thus demonstrate the suitability of the applied synthesis protocol for the SPPS of such long affibody sequences.

It can, therefore, be concluded that both synthesis routes—using conventional mechanical shaking or ultrasound-assisted SPPS—yielded the desired affibody, Z_EGFR:1907_, without significant differences in product composition or achievable isolated yields.

This seems surprising at first glance, as we [6,26] as well as other groups [20,25] have previously shown that ultrasonication is able to significantly increase the coupling efficiency of amino acid building blocks during Fmoc strategy-based SPPS on peptides of up to 44 amino acids in length. Using this method, comparable or even considerably improved synthesis results were found when the excesses of reagents or the coupling times per amino acid building block were reduced, demonstrating the higher reaction efficacy as a result of ultrasonication.

As an explanation for the observation made here that ultrasound assistance was not able to improve product formation during the synthesis of Z_EGFR:1907_ compared to conventional mechanical shaking, it could be suggested that ultrasonication usually compensates for non-optimal reaction conditions (reduced amounts of reactants and less time available for reaction), whereby comparable results to conventional protocols can be obtained. In contrast, under optimal reaction conditions using standard high reactant excesses and prolonged reaction times with increasing sequence length, the results presented here seem to indicate that no further improvement is achieved by ultrasonication, as an increase in reaction efficiency is no longer able to come into effect. If this assumption is correct, ultrasound assistance could demonstrate a measurable effect in SPPS whereby the application of reduced reaction times and reagent excesses by allowing the same yield and purity of the obtained affibody products to be maintained, in contrast to mechanical shaking. This would represent a great advantage in terms of the overall efficiency, productivity, and sustainability of SPPS with large peptide molecules. However, these parameters were not the focus of this work; rather, the aim was to determine whether it is possible to obtain affibodies of a relatively high purity and yield through chemical synthesis to make them available for the development of radiopharmaceuticals. We expected that ultrasound assistance could make a significant contribution to this, but were not able to show this effect; rather, we found that the resin has a stronger influence on synthesis outcome. As a result, we were able to show that it is possible to obtain affibodies under standard optimal Fmoc-based SPPS conditions, hopefully encouraging radiopharmaceutical development based on this highly favorable class of biomolecules.

## 3. Conclusions

The aim of the present systematic study was to investigate the extent to which ultrasound-assisted Fmoc-strategy-based solid-phase peptide synthesis is superior to conventional protocols that apply mechanical shaking for the synthesis of very long peptides such as affibodies, investigated here using Z_EGFR:1907_ as an example.

It was found that the solid support used for the synthesis had a considerable influence on the obtained results, whereas conventional and ultrasound-assisted syntheses yielded comparable results with analogous composition of the raw products using a well-suited solid support, reactant excesses, and coupling times. The Z_EGFR:1907_ affibody was obtained in very satisfactory isolated yields, regardless of the use of ultrasound, which indicates that under the conditions applied here, the choice of resin used had a greater influence on the synthesis results for Z_EGFR:1907_ than the increase in efficiency of the coupling of the individual amino acid building blocks using ultrasonication.

Thus, great attention should be paid to the choice of a suitable solid support for affibody synthesis by Fmoc-strategy-based SPPS, enabling this highly interesting class of biomolecules to be obtained in good yields and to bring them more into the focus of radiopharmaceutical research.

## 4. Materials and Methods

Chemicals. All chemicals were purchased from commercial sources in analytical-grade quality and used without further purification unless otherwise stated. Fmoc- and side-chain-protected amino acids, NovaSyn TGR R resin (loading 0.19 mmol/g), Sieber TG (loading 0.21 mmol/g), and Rink (loading 0.36 mmol/g) resins were purchased from NovaBiochem (Darmstadt, Germany). Diethyl ether, dimethylformamide (DMF), piperidine, 2-(1*H*-benzotriazol-1-yl)-1,1,3,3-tetramethyluronium hexafluorophosphate (HBTU), trifluoroacetic acid (TFA), and water were obtained from Carl Roth (Karlsruhe, Germany). Acetonitrile (MeCN) and Dichloromethane (DCM) were obtained from Häberle Labortechnik (Lonsee-Ettlenschieß, Germany), and *N,N*-diisopropylethylamine (DIPEA) and triisopropylsilane (TIS) from Sigma Aldrich (Taufkirchen, Germany).

Instrumentation. HPLC: Analytical and semipreparative HPLC analyses were conducted utilizing a Dionex UltiMate 3000 system (Dreieich, Germany) together with a Chromolith SemiPrep (RP-18e, 100-10 mm, Merck, Germany) column and the data were analyzed using Chromeleon software (Version 6.80). MALDI-TOF MS: Matrix-Assisted Laser Desorption/Ionization (MALDI) time-of-flight mass spectra were obtained by utilizing a Bruker Daltonics Microflex spectrometer (Bremen, Germany), linear acquisition mode, positive ion source, and 200 shots per spot. α-Cyano-4-hydroxycinnamic acid (α-CHCA) was chosen as a matrix and the dried-droplet method was used for sample preparation on a micro scout target (MSP 96 target polished steel BC, Bruker Daltonics, Bremen, Germany). The data were recorded with flexControl Version 3.3 and analyzed with flexAnalysis Version 3.3 software. HR-ESI-MS: For high-resolution electrospray ionization mass spectrometry (HR-ESI-MS), a Thermo Finnigan LTQ FT Ultra Fourier Transform Ion Cyclotron Resonance (Dreieich, Germany) mass spectrometer was used. The resolution was adjusted to 100.000 at *m*/*z* 400. Depending on the sample, a mass range of 50–2000 u was chosen. Ultrasonic bath: Ultrasound-assisted syntheses were performed in a Bandelin Sonorex Super RK 225 H ultrasonic bath (Berlin, Germany) with the temperature of the water kept at 30–45 °C. The temperature range resulted from the water in the ultrasonic bath warming up during the individual coupling reactions from around 30 °C at the beginning to around 45 °C after 60 min of coupling time. The water was exchanged after each coupling step and during the 60 minutes coupling steps after half the time.

Peptide Syntheses. All peptidic compounds were assembled on a commercially available resin using standard Fmoc-strategy-based solid-phase peptide synthesis (SPPS) protocols [6,7,26]. Conventional syringes (10 mL, HSW, Tuttlingen, Germany) equipped with two layers of 35 μm porous high-density polyethylene frits (Reichelt Chemietechnik, Heidelberg, Germany) were used as reaction chambers, with the resin placed between the plunger and frit. Directly before coupling of the first amino acid, the resin (0.19 mmol/g, 555 mg, 105.5 µmol) was swollen for 45 min in DCM and, afterwards, washed thrice with DMF. Coupling reactions were carried out in DMF for 30 min (amino acid 1–20), 45 min (amino acid 21–40), or 60 min (amino acid 41–58) using conventional mechanical shaking or ultrasound using a conventional ultrasonic bath at 30–45 °C. For building block conjugation, 4 equiv. of the respective amino acid was taken together with 3.9 equiv. of HBTU as the coupling reagent and 4 equiv. of DIPEA as the base. The removal of Fmoc-protecting groups was performed with 50% piperidine in DMF (*v*:*v*) (2 and 5 min). Acid-labile protecting groups were removed while simultaneously cleaving the respective compound from the resin with a mixture of TFA, TIS, and H_2_O (95:2.5:2.5, *v*:*v*:*v*) for 3 h at room temperature; afterwards. The resin was washed with TFA (3 × 5 mL) to maximize product recovery. Volatile materials were evaporated and the crude products were dissolved in a mixture of H_2_O:MeCN + 0.1% TFA 1:1 (*v*:*v*) and analyzed and purified by semipreparative HPLC using the gradients of 10–60% and 0–100% MeCN over 8 minutes (in this manuscript, only chromatograms using the 10–60% gradient are shown unless otherwise stated). The final products were obtained in yields of 20.5 ± 6.1% for ultrasound-assisted syntheses and 24.4 ± 0.6% for conventional syntheses after lyophilization.

MALDI-TOF-MS (*m*/*z*, matrix: α-cyano-4-hydroxycinnamic acid) and HR-ESI-MS (*m*/*z*) data for **1**–**6**:

**1**: MALDI-TOF-MS (*m*/*z*) using α-cyano-4-hydroxycinnamic acid as the matrix substance [M + H]^+^ (calculated): 1111.24 (1111.6582). HR-ESI-MS (*m*/*z*) [M + H]^+^ (calculated): 1111.6568 (1111.6582), [M + H]^2+^ (calculated): 556.3326 (556.3328), [M + H]^3+^ (calculated): 371.2242 (371.2243).

**2**: MALDI-TOF-MS (*m*/*z*) using α-cyano-4-hydroxycinnamic acid as the matrix substance [M + H]^+^ (calculated): 2096.24 (2096.1458), [M + H]^2+^ (calculated): 1048.66 (1048.5766). HR-ESI-MS (*m*/*z*) [M + H]^2+^ (calculated): 1048.5761 (1048.5766), [M + H]^3+^ (calculated): 699.3873 (699.3868).

**3**: MALDI-TOF-MS (*m*/*z*) using α-cyano-4-hydroxycinnamic acid as the matrix substance [M + H]^+^ (calculated): 3125.37 (3124.6637), [M + H]^2+^ (calculated): 1562.94 (1562.8355). HR-ESI-MS (*m*/*z*) [M + H]^2+^ (calculated): 1562.8401 (1562.8355), [M + H]^3+^ (calculated): 1042.2270 (1042.2261), [M + H]^4+^ (calculated): 781.9231 (781.9214).

**4**: MALDI-TOF-MS (*m*/*z*) using α-cyano-4-hydroxycinnamic acid as the matrix substance [M + H]^+^ (calculated): 4282.84 (4279.2180), [M + H]^2+^ (calculated): 2140.26 (2140.1126). HR-ESI-MS (*m*/*z*) [M + H]^3+^ (calculated): 1427.0821 (1427.0775), [M + H]^4+^ (calculated): 1070.5625 (1070.5600).

**5**: MALDI-TOF-MS (*m*/*z*) using α-cyano-4-hydroxycinnamic acid as the matrix substance [M + H]^+^ (calculated): 5575.11 (5565.8046), [M + H]^2+^ (calculated): 2783.20 (2783.4060). HR-ESI-MS (*m*/*z*) [M + H]^3+^ (calculated): 1855.9543 (1855.9397), [M + H]^4+^ (calculated): 1392.2111 (1392.2066), [M + H]^5+^ (calculated): 1113.9681 (1113.9667).

**6**: MALDI-TOF-MS (*m*/*z*) using α-cyano-4-hydroxycinnamic acid as the matrix substance [M + H]^+^ (calculated): 6554.55 (6540.2867), [M + H]^2+^ (calculated): 3269.68 (3270.6470). HR-ESI-MS (*m*/*z*) [M + H]^4+^ (calculated): 1635.8336 (1635.8272), [M + H]^5+^ (calculated): 1308.8662 (1308.8632), [M + H]^6+^ (calculated): 1090.8874 (1090.8872).

## Figures and Tables

**Table 1:** Comparative results of the HPLC analyses regarding the proportion of product in the crude peptide mixtures applying conventional (CV) or ultrasound-assisted (US) coupling protocols as well as mass spectrometry analyses of the peptide fragments **1**–**5** and the full peptide sequence **6** using MALDI-MS and HR-ESI-MS (ESI: electrospray ionization).

Peptide Fragment	Proportion of Product in the Crude Peptide Mixture [%]	Ion [*m*/*z*]	Calculated Exact Mass [g/mol]	Detected MALDI Mass [g/mol]	Detected HR-ESI Mass [g/mol]
**1**	94.7 ± 3.0 (US) 91.4 ± 2.2 (CV)	[M + H]^+^	1111.6582	1111.24	1111.6568
		[M + H]^2+^	556.3328	–	556.3326
		[M + H]^3+^	371.2243	–	371.2242
**2**	83.8 ± 1.3 (US) 82.5 ± 2.9 (CV)	[M + H]^+^	2096.1458	2096.24	–
		[M + H]^2+^	1048.5766	1048.66	1048.5761
		[M + H]^3+^	699.3868	–	699.3873
**3**	80.7 ± 2.5 (US) 81.2 ± 2.4 (CV)	[M + H]^+^	3124.6637	3125.37	–
		[M + H]^2+^	1562.8355	1562.94	1562.8401
		[M + H]^3+^	1042.2261	–	1042.2270
		[M + H]^4+^	781.9214	–	781.9231
**4**	45.5 ± 4.3 (US) 44.2 ± 0.1 (CV)	[M + H]^+^	4279.2180	4282.84	–
		[M + H]^2+^	2140.1126	2140.26	–
		[M + H]^3+^	1427.0775	–	1427.0821
		[M + H]^4+^	1070.5600	–	1070.5625
**5**	39.3 ± 0.7 (US) 36.6 ± 0.3 (CV)	[M + H]^+^	5565.8046	5575.11	–
		[M + H]^2+^	2783.4060	2783.20	–
		[M + H]^3+^	1855.9397	–	1855.9543
		[M + H]^4+^	1392.2066	–	1392.2111
		[M + H]^5+^	1113.9667	–	1113.9681
**6** 25.3 ± 3.2 (US) 24.8 ± 2.4 (CV) 20.5 ± 6.1% ^1^ (US) 24.4 ± 0.6% ^1^ (CV)		[2M+H]^+^	13,079.5668	13,079.5569 ^2^	–
		[M + H]^+^	6540.2867	6540.2877 ^2^	–
		[M + H]^2+^	3270.6470	3270.6473 ^2^	–
		[M + H]^3+^	2180.7671	–	–
		[M + H]^4+^	1635.8272	–	1635.8336
		[M + H]^5+^	1308.8632	–	1308.8662
		[M + H]^6+^	1090.8872	–	1090.8874

^1^ isolated yields after purification and lyophilization; ^2^ HR-MALDI results.

## Data Availability

Data is contained within the article and Supplementary Materials.

## Supplementary Materials

The following supporting information can be downloaded at: https://www.mdpi.com/article/10.3390/ph17101280/s1, Figure S1: Direct comparison of HPLC chromatograms of affibody raw product mixtures obtained by applying a Sieber TG resin as the solid support. Representative MALDI-MS and HR-ESI-MS spectra of peptide fragments **1**–**5** and the complete affibody peptide sequence **6**.
