# Supplementary material for: Ultrasound-Assisted Solid-Phase Affibody Synthesis Using ZEGFR:1907 as an Example—Superior to the Conventional Protocol?
Source: Pharmaceuticals (Basel). 2024 Sep 27;17(10):1280. doi: 10.3390/ph17101280 (PMC11510254; doi:10.3390/ph17101280)

Supplementary Material for

Ultrasound-Assisted Solid-Phase Affibody Synthesis using $Z_{EGFR:1907}$ as an Example — Superior to the Conventional Protocol?

Marie Prochiner [1,2], Benedikt Judmann [1,2], Alina Ruder [1,2], Björn Wängler [2,3], Ralf Schirrmacher [4] and Carmen Wängler [1,2,*]

[1] Biomedical Chemistry, Clinic of Radiology and Nuclear Medicine, Medical Faculty Mannheim, Heidelberg University, Theodor-Kutzer-Ufer 1-3, 68167 Mannheim, Germany; marie.prochiner@stud.uni-heidelberg.de (M.P.); benedikt.judmann@medma.uni-heidelberg.de (B.J.); ruderal102168@th-nuernberg.de (A.R.)

[2] Research Campus M²OLIE, Medical Faculty Mannheim, Heidelberg University, Theodor-Kutzer-Ufer 1-3, 68167 Mannheim, Germany;

[3] Molecular Imaging and Radiochemistry, Clinic of Radiology and Nuclear Medicine, Medical Faculty Mannheim, Heidelberg University, Theodor-Kutzer-Ufer 1-3, 68167 Mannheim, Germany; bjoern.waengler@medma.uni-heidelberg.de (B.W.)

[4] Division of Oncological Imaging, Department of Oncology, University of Alberta, Edmonton, AB T6G 1Z2, Canada; schirrma@ualberta.ca (R.S.)

[*] Correspondence: carmen.waengler@medma.uni-heidelberg.de

Content:

Fig. S1: Direct comparison of HPLC chromatograms of affibody raw product mixtures obtained applying a Sieber TG resin as the solid support at the stage of 55 amino acids using conventional mechanical agitation or ultrasonication.	page 2
Representative MALDI-MS, HR-ESI-MS and HR-MALDI spectra of peptide fragments **1** – **5** and the complete affibody peptide sequence **6**.	page 2

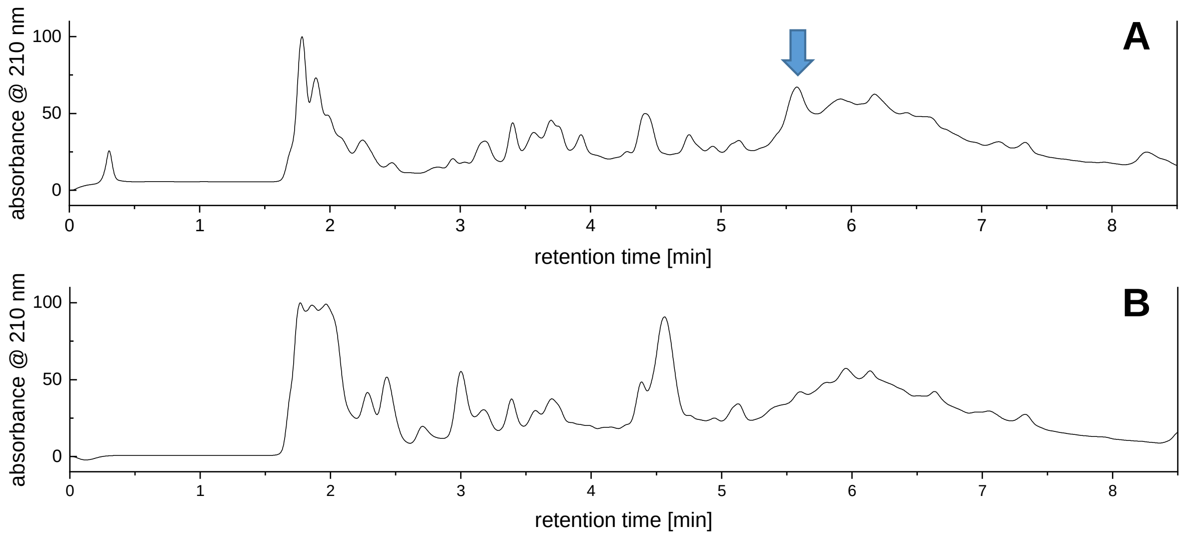

Fig. S1: Direct comparison of HPLC chromatograms of affibody raw product mixtures obtained applying a Sieber TG resin as the solid support at the stage of 55 amino acids using conventional mechanical agitation (**A**) or ultrasonication (**B**) otherwise applying analogous reaction conditions. The analyses were carried out using a gradient of 20 – 60% MeCN over 8 min on a Chromolith SemiPrep (RP-18e, 100-10 mm) column. The intended product was found for the peak marked with the arrow.

Representative MALDI-MS, HR-ESI-MS and HR-MALDI spectra of peptide fragments **1** – **5** and the complete affibody peptide sequence **6**.

Fragment **1**

MALDI

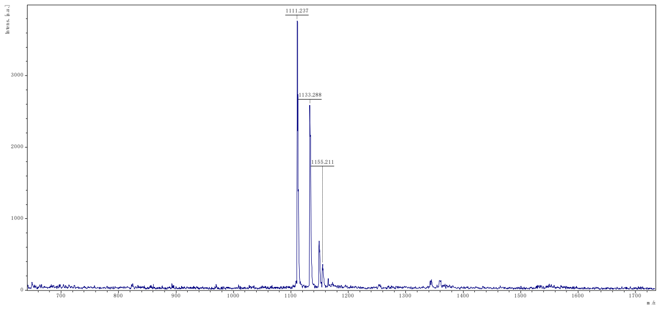

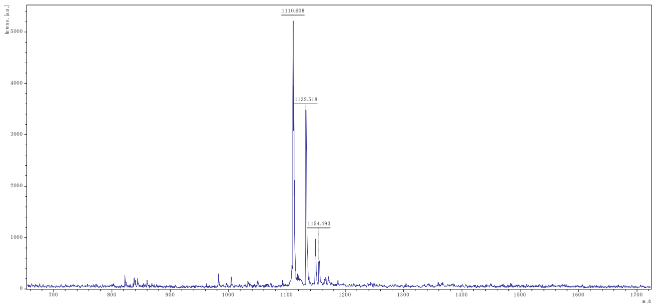

HR-ESI

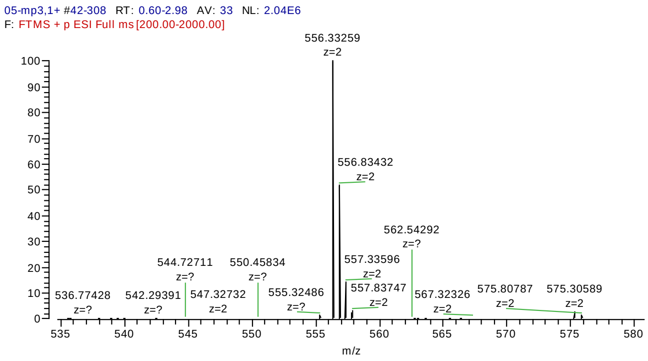

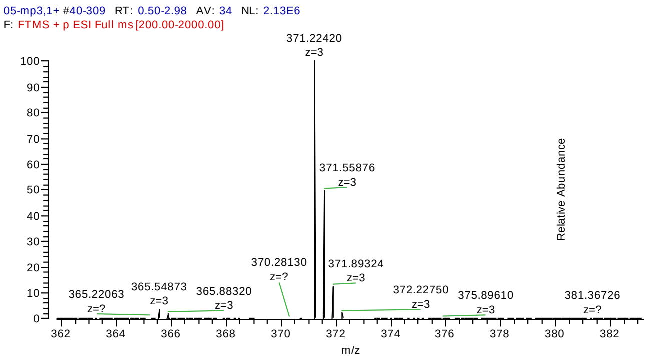

Fragment **2**

MALDI

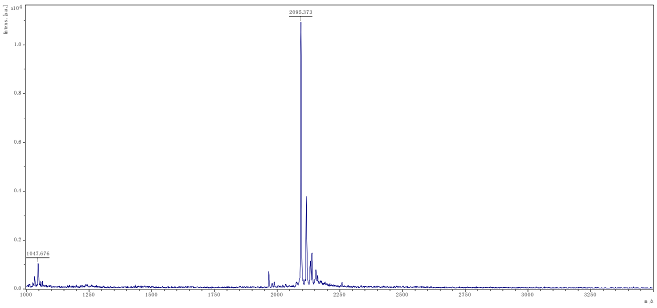

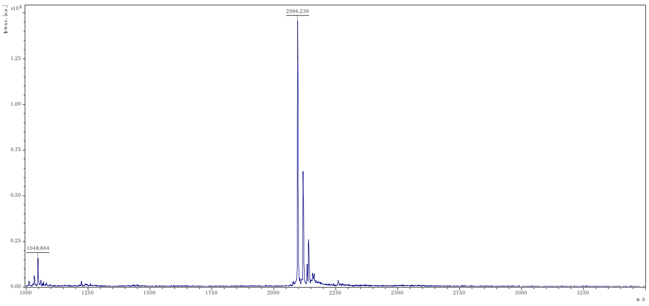

HR-ESI

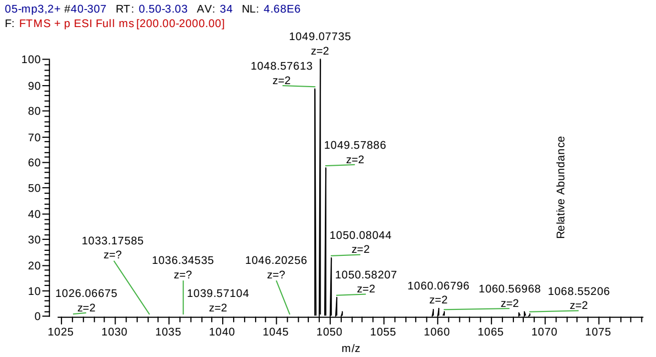

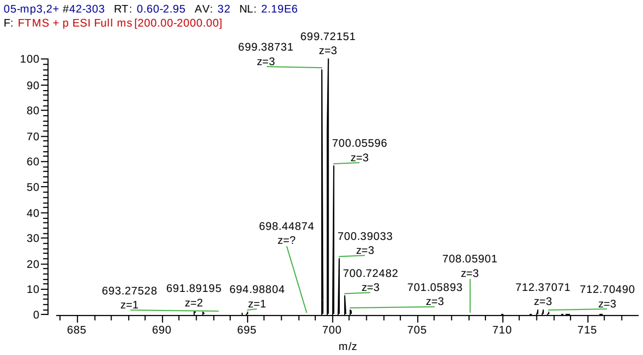

Fragment **3**

MALDI

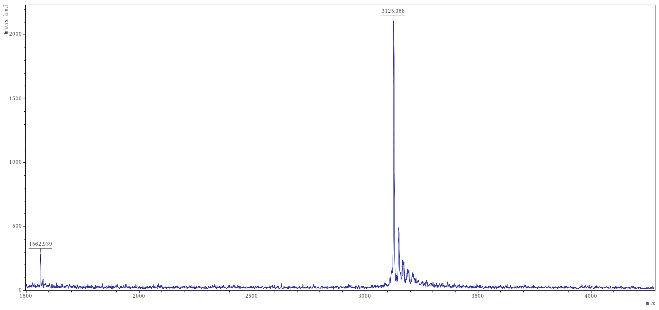

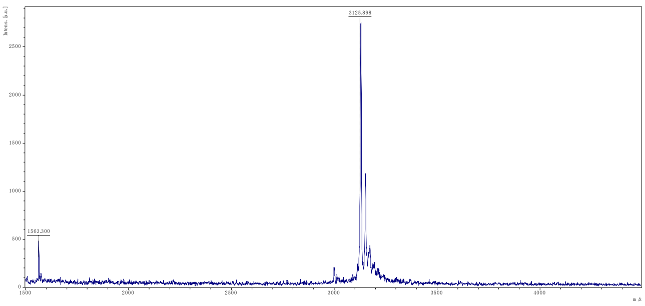

HR-ESI

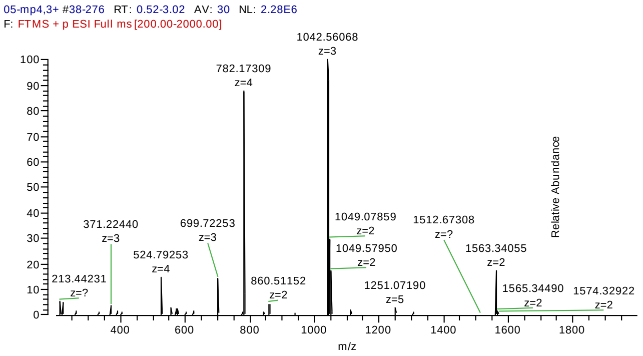

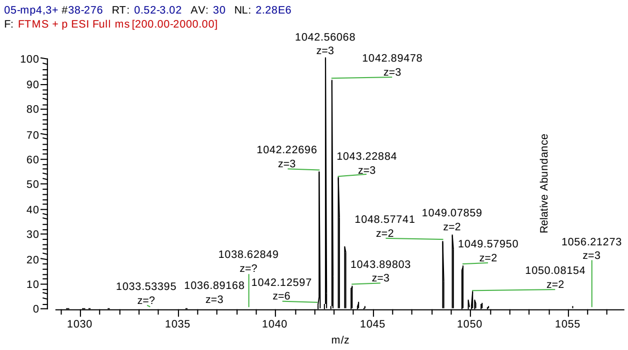

Fragment **4**

MALDI

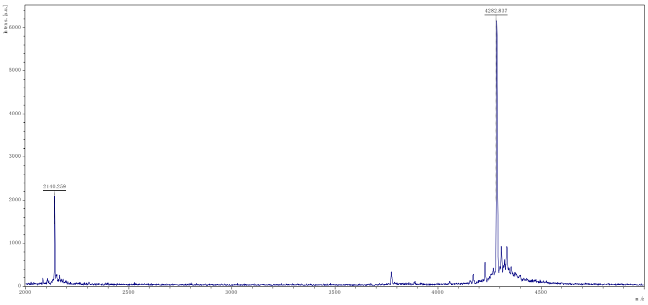

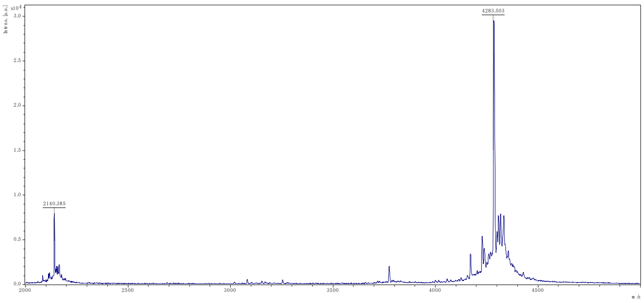

HR-ESI

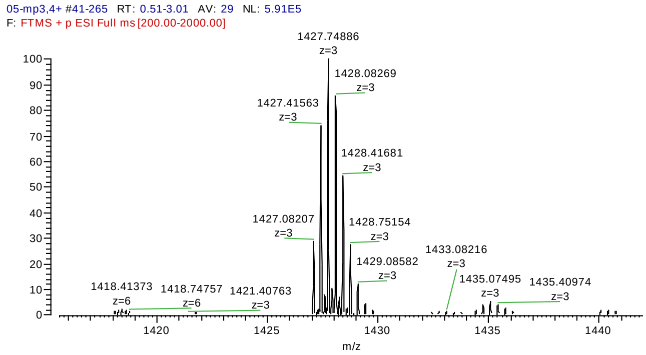

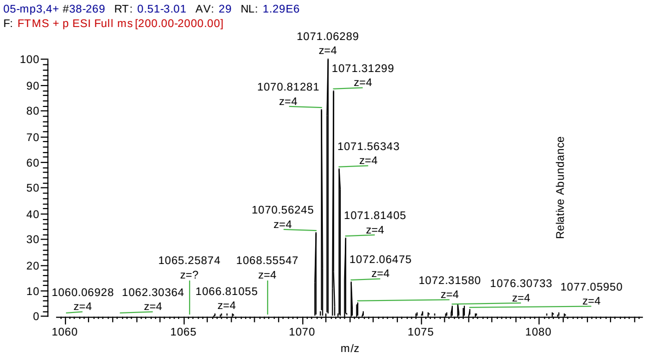

Fragment **5**

MALDI

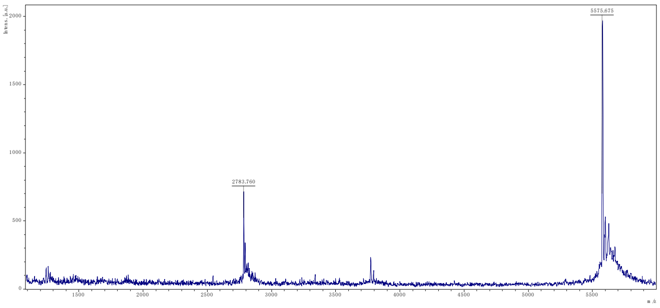

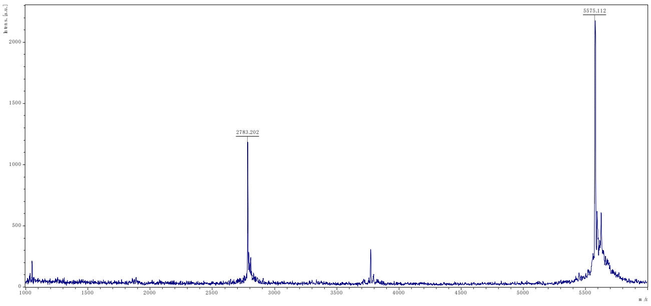

HR-ESI

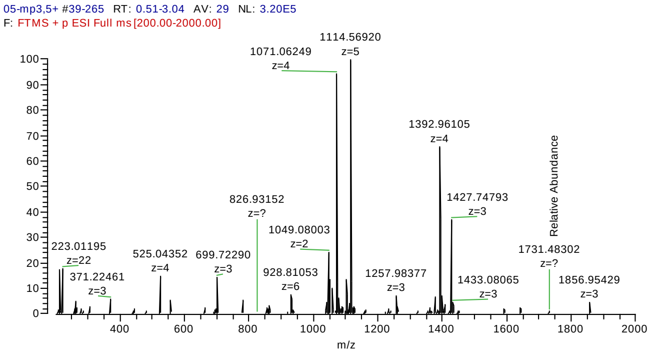

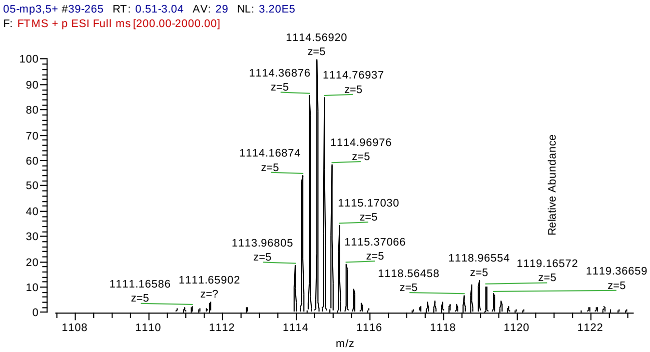

Full-length affibody **6**

MALDI

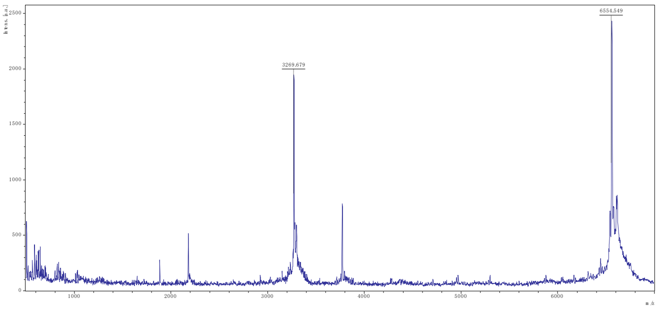

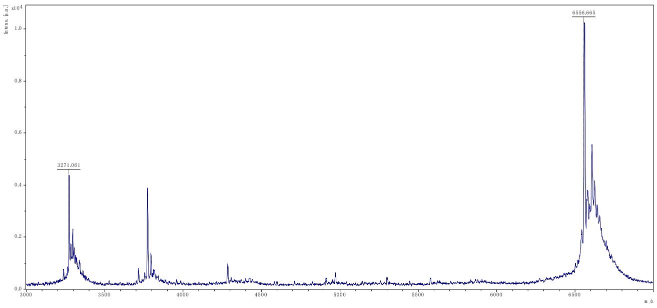

HR-ESI

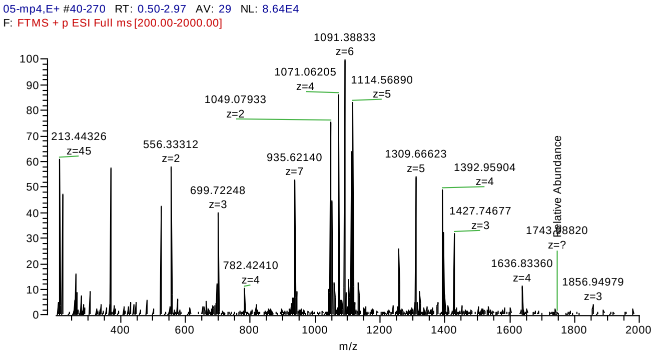

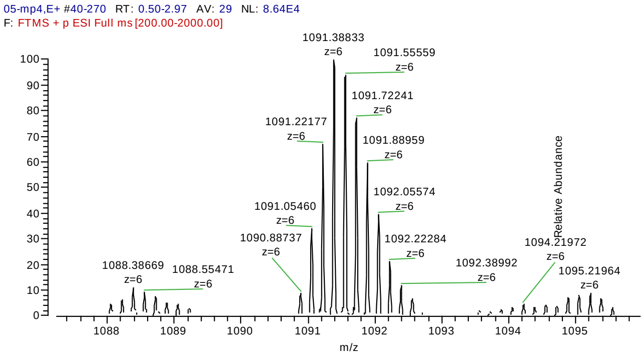

HR-MALDI

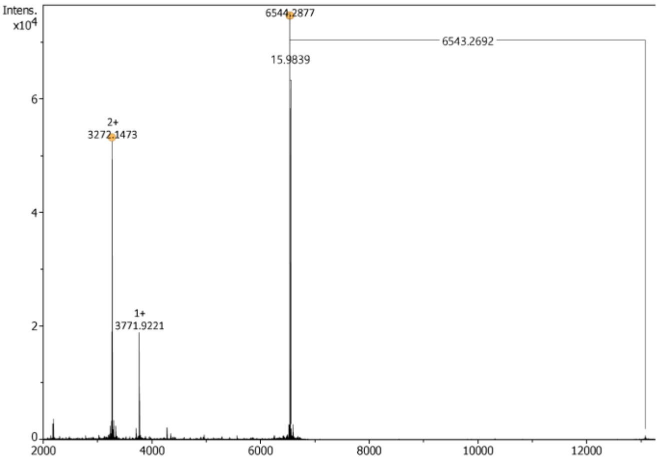

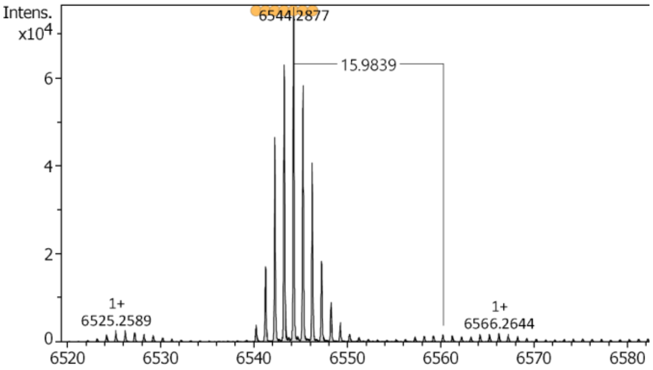

Supplement: Supplementary file 1 [file pharmaceuticals-17-01280-s001.zip › pharmaceuticals-3208977-supplementary.pdf]
